# Influence of the Intestinal Microbiota on Colonization Resistance to *Salmonella* and the Shedding Pattern of Naturally Exposed Pigs

**DOI:** 10.1128/mSystems.00021-19

**Published:** 2019-04-23

**Authors:** Héctor Argüello, Jordi Estellé, Finola C. Leonard, Fiona Crispie, Paul D. Cotter, Orla O’Sullivan, Helen Lynch, Kavita Walia, Geraldine Duffy, Peadar G. Lawlor, Gillian E. Gardiner

**Affiliations:** a Teagasc, Food Research Centre, Ashtown, Dublin, Ireland; b Grupo de Genómica y Mejora Animal, Departamento de Genética, Facultad de Veterinaria, Universidad de Córdoba, Córdoba, Spain; c GABI, INRA, AgroParisTech, Université Paris-Saclay, Jouy-en-Josas, France; d School of Veterinary Medicine, University College Dublin, Dublin, Ireland; e Teagasc, Food Research Centre, Fermoy, Cork, Ireland; f APC Microbiome Ireland, Cork, Ireland; g Teagasc, Pig Development Department, Animal and Grassland Research and Innovation Centre, Moorepark, Fermoy, Cork, Ireland; h Department of Science, Waterford Institute of Technology, Waterford, Ireland; University of Southampton

**Keywords:** microbiome, colonization resistance, metagenome, pathogen, pig

## Abstract

*Salmonella* colonization and infection in production animals such as pigs are a cause for concern from a public health perspective. Variations in susceptibility to natural infection may be influenced by the intestinal microbiota. Using 16S rRNA compositional sequencing, we characterized the fecal microbiome of 15 weaned pigs naturally infected with *Salmonella* at 18, 33, and 45 days postweaning. Dissimilarities in microbiota composition were analyzed in relation to *Salmonella* infection status (infected, not infected), serological status, and shedding pattern (nonshedders, single-point shedders, intermittent-persistent shedders). Global microbiota composition was associated with the infection outcome based on serological analysis. Greater richness within the microbiota postweaning was linked to pigs being seronegative at the end of the study at 11 weeks of age. Members of the *Clostridia*, such as *Blautia*, *Roseburia*, and *Anaerovibrio*, were more abundant and part of the core microbiome in nonshedder pigs. Cellulolytic microbiota (*Ruminococcus* and *Prevotella*) were also more abundant in noninfected pigs during the weaning and growing stages. Microbial profiling also revealed that infected pigs had a higher abundance of *Lactobacillus* and *Oscillospira*, the latter also being part of the core microbiome of intermittent-persistent shedders. These findings suggest that a lack of microbiome maturation and greater proportions of microorganisms associated with suckling increase susceptibility to infection. In addition, the persistence of *Salmonella* shedding may be associated with an enrichment of pathobionts such as *Anaerobiospirillum*. Overall, these results suggest that there may be merit in manipulating certain taxa within the porcine intestinal microbial community to increase disease resistance against *Salmonella* in pigs.

**IMPORTANCE** *Salmonella* is a global threat for public health, and pork is one of the main sources of human salmonellosis. However, the complex epidemiology of the infection limits current control strategies aimed at reducing the prevalence of this infection in pigs. The present study analyzes for the first time the impact of the gut microbiota in *Salmonella* infection in pigs and its shedding pattern in naturally infected growing pigs. Microbiome (16S rRNA amplicon) analysis reveals that maturation of the gut microbiome could be a key consideration with respect to limiting the infection and shedding of *Salmonella* in pigs. Indeed, seronegative animals had higher richness of the gut microbiota early after weaning, and uninfected pigs had higher abundance of strict anaerobes from the class *Clostridia*, results which demonstrate that a fast transition from the suckling microbiota to a postweaning microbiota could be crucial with respect to protecting the animals.

## Abstract (precis)

*Salmonella* is a global threat for public health, and pork is one of the main sources of human salmonellosis. However, the complex epidemiology of the infection limits current control strategies aimed at reducing the prevalence of this infection in pigs. The present study analyzes for the first time the impact of the gut microbiota in *Salmonella* infection in pigs and its shedding pattern in naturally infected growing pigs. Microbiome (16S rRNA amplicon) analysis reveals that maturation of the gut microbiome could be a key consideration with respect to limiting the infection and shedding of *Salmonella* in pigs. Indeed, seronegative animals had higher richness of the gut microbiota early after weaning, and uninfected pigs had higher abundance of strict anaerobes from the class *Clostridia*, results which demonstrate that a fast transition from the suckling microbiota to a postweaning microbiota could be crucial with respect to protecting the animals.

## INTRODUCTION

*Salmonella* species is a ubiquitous enterobacterium which colonizes the intestine of animals (1). Nontyphoidal serovars such as Salmonella enterica serovar Typhimurium and Salmonella enterica serovar Enteritidis, which are frequently present in the gastrointestinal tract of production animals, are a major source of human salmonellosis (2), and recent studies identify pork products as one of the main sources of infection (3). Pigs are a natural host for *Salmonella* (4), and infection can occur at any production stage (5). With the exception of infections caused by Salmonella enterica serovar Choleraesuis, the serovar which is host adapted to pigs (6), the course of clinical infection is restricted to intestinal disease and is usually subclinical (7). *Salmonella* infection in swine is characterized by an early acute phase in which the pathogen is shed in relatively high concentrations in the feces (8). This then progresses to intermittent shedding or carriage with reactivation of shedding under adverse circumstances (9).

*Salmonella* epidemiological studies demonstrate that infected and noninfected pigs cohabit within the same herd (10, 11). Differences in disease outcome are also observed in animals monitored during field trials (12, 13). This phenomenon may reflect individual variability in susceptibility in pigs exposed to *Salmonella* under the same environmental conditions. Furthermore, infected animals exhibit variations in the concentration and duration of pathogen excretion in the feces (5, 8, 14). This could, at least in part, be due to colonization resistance, a concept first proposed in the 1950s (15, 16) but which may be relevant to the interpretation of recent studies investigating the influence of the microbiome on disease outcomes (17). The principle underlying colonization resistance is that normal gut symbionts can form a barrier in the gastrointestinal tract, which limits the invasion of nonnative bacteria such as pathogens (18–20).

The observed differences in gastrointestinal colonization of pigs by *Salmonella* under natural conditions, including variations in shedding pattern and resistance to colonization, could therefore be ascribed, at least partially, to the resident microbiota of the host. Recently, high-throughput sequencing has enabled metagenomic cataloguing of pig intestinal samples, thereby providing insights into the microbial species present within the porcine intestinal tract (21–23). This has revolutionized our ability to study the gut microbiome, under different conditions, including deliberate *Salmonella* infection (24, 25).

In this paper, we present a novel study of the fecal microbiome of naturally infected weaned pigs from a *Salmonella*-positive herd, in which apparent differences in susceptibility to *Salmonella* infection and divergence in shedding pattern among penmates were observed. The overall aim was to identify, for the first time, particular groups of bacteria associated with the outcome of *Salmonella* infection in pigs naturally infected with the pathogen.

## RESULTS

### Diversity of the microbiota in pigs categorized according to *Salmonella* infection-associated variables.

**FIG 1:**
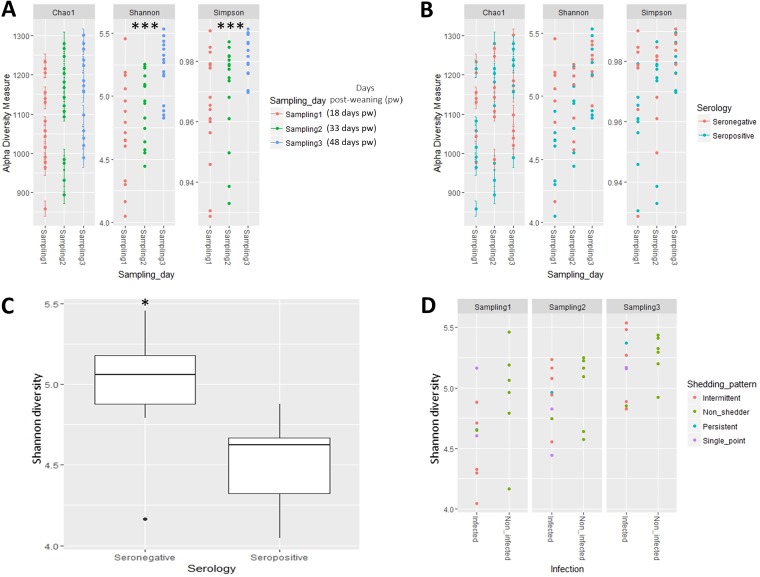
Alpha-diversity of the fecal microbiota of weaned pigs naturally infected with *Salmonella* or noninfected as determined by Shannon, Simpson, and Chao1 indices at the different sampling time points postweaning (A, B, and D) and according to *Salmonella* serology (seronegative or seropositive) (B). (C) Difference in Shannon index between seronegative and seropositive pigs (*P* < 0.01) at fecal sampling 1 (18 days pw). (D) Shannon index of fecal samples according to *Salmonella* infection status (infected versus noninfected pigs) and shedding pattern. Significant differences are denoted as follows: *P < *0.05, *; *P < *0.01, **; and *P < *0.001, ***.

After filtering, 16S rRNA amplicon sequences were assigned to 1,493 taxa across seven taxonomic ranks. Alpha-diversity of the fecal microbiota was measured using three different estimators (Fig. 1; see also Table S1 in the supplemental material). The Shannon and Simpson indices (*P < *0.01) but not Chao1 (*P = *0.103) revealed progressive increases in diversity at each sampling time point (Fig. 1A). Analysis of variables associated with *Salmonella* (infection status, serology, shedding pattern and shedding group) and alpha-diversity measures suggested a link between serological status and Shannon index value (*P = *0.0653, Fig. 1B). This trend was influenced by the significant differences observed at sampling 1 (18 days postweaning [pw]) when the Shannon index was clearly higher in seronegative pigs (*P < *0.05, Fig. 1C), a result not observed at sampling 2 or sampling 3 (Fig. 1B; Table S1). No differences in alpha-diversity of the fecal microbiota were observed for infection status, *Salmonella* shedding pattern (Fig. 1D), or shedding group during the course of the study (Fig. S1).

10.1128/mSystems.00021-19.1 FIG S1 Alpha diversity of the fecal microbiota of weaned pigs naturally infected with *Salmonella* or noninfected as determined by Shannon, Simpson, and Chao1 indices according to *Salmonella* infection (infected and noninfected) (A), shedding pattern (nonshedder, single-point shedder, intermittent shedder, and persistent shedder) (B), and shedding groups (A, B, and C) (C). Download FIG S1, TIF file, 1.1 MB. Copyright © 2019 Argüello et al. 2019 Argüello et al. This content is distributed under the terms of the Creative Commons Attribution 4.0 International license.

10.1128/mSystems.00021-19.2 TABLE S1 Alpha-diversity estimations (observed OTUs, Shannon, inverted Simpson, Simpson, Chao1) and *P* values obtained when alpha-diversity by the estimators selected was compared by the factors under study. Download Table S1, XLSX file, 0.02 MB. Copyright © 2019 Argüello et al. 2019 Argüello et al. This content is distributed under the terms of the Creative Commons Attribution 4.0 International license.

### Influence of study variables on sample ordination.

**FIG 2:**
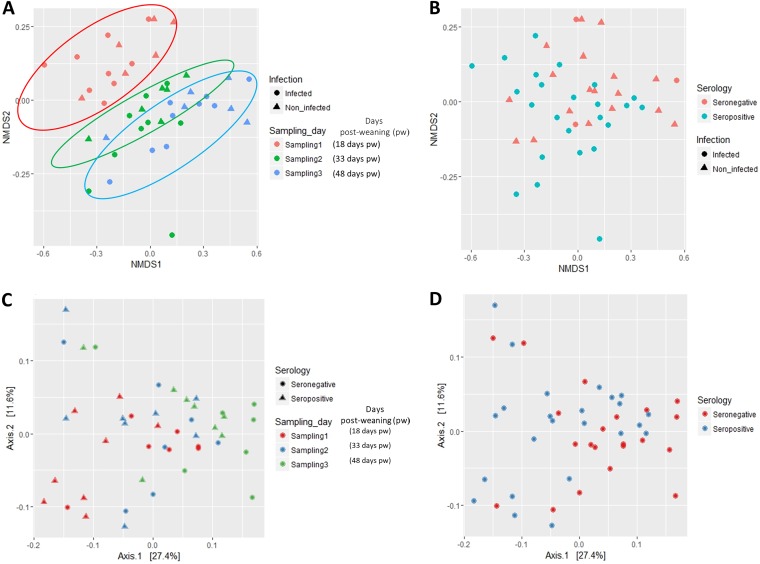
Ordination analysis of fecal samples obtained from weaned pigs naturally infected with *Salmonella* or noninfected. (A and B) Effect of sampling time point (A) and *Salmonella* serology (B) on Bray-Curtis distance of samples represented by nonmetric multidimensional scaling (NMDS). (C and D) The same factors analyzed by the weighted UniFrac method.

Bray-Curtis (Fig. 2A and B) and weighted UNIFRAC distance analysis (Fig. 2C and D) provided consistent insights in relation to the ordination of pig fecal samples according to the different factors under study. Fitting these environmental factors (infection status, shedding group, serology, pig or sampling time point, feed type) revealed an influence of sampling time point (*P < *0.001) (linked in part to the change in diet [*P* < 0.01]) and serology (*P = *0.035) variables on the Bray-Curtis ordination of samples (Table S2). The strong influence of sampling time point on the ordination of samples was corroborated by performing a multivariate ANOVA based on dissimilarities (Table S3; *P < *0.01). Neither *Salmonella* infection status nor shedding group influenced ordination of the pig fecal samples (*P > *0.05; Table S3).

10.1128/mSystems.00021-19.3 TABLE S2 Analysis (*envfit* function of Vegan) of the influence of different factors on the ordination of samples. Significance was established at α = 0.05. Download Table S2, XLSX file, 0.01 MB. Copyright © 2019 Argüello et al. 2019 Argüello et al. This content is distributed under the terms of the Creative Commons Attribution 4.0 International license.

10.1128/mSystems.00021-19.4 TABLE S3 Results of the permutation multivariate ANOVA performed in the ordination analysis. Download Table S3, XLSX file, 0.01 MB. Copyright © 2019 Argüello et al. 2019 Argüello et al. This content is distributed under the terms of the Creative Commons Attribution 4.0 International license.

### Core microbiome analysis.

**FIG 3:**
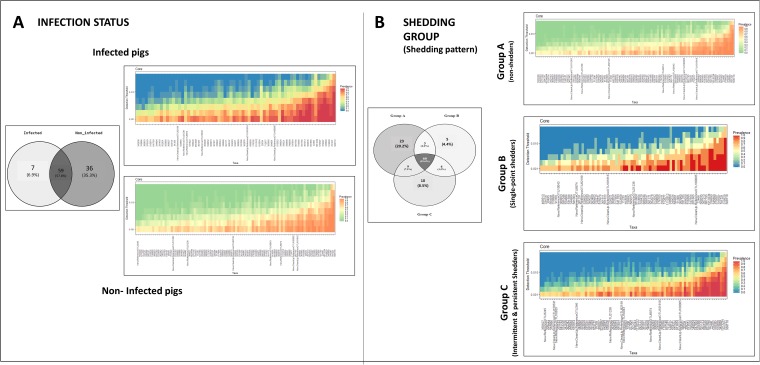
Analysis of the core microbiome associated with *Salmonella* infection in weaned pigs. Core microbiome of pigs categorized according to *Salmonella* infection status (A) and *Salmonella* shedding, i.e., group A (nonshedders), group B (single-point shedders), and group C (intermittent/persistent shedders) (B). The number and percentage of OTUs overlapping between categories are also shown (in Venn diagrams).

The core microbiome was established for each category within the variables “infection status” and “shedding group” (Fig. 3). Data were not split by sampling time point, in order to obtain a complete picture of the bacteria defining the core microbiome of each group, irrespective of any modifications occurring over time. *Salmonella-*infected and noninfected pigs shared more than half of the operational taxonomic units (OTUs) included in the core microbiome, while 7 and 36 OTUs were present only within the core microbiome of infected and noninfected pigs, respectively (Fig. 3A). The seven unique OTUs found in the infected pigs belonged to the genera *Lachnospira* and *Prevotella*. Operational taxonomic units from these two genera were also present in the core microbiome of noninfected pigs. In contrast, the core microbiome of noninfected pigs included OTUs from *Phascolarctobacterium*, *Roseburia*, and *Blautia*, genera which were not present within the core microbiome of infected pigs. The core microbiome was also established for pigs categorized according to *Salmonella* shedding. Most of the OTUs were shared among the three shedding categories (A, B, and C). Twenty-three OTUs were part of the core microbiome in nonshedder pigs only (group A), 5 OTUs were unique to single-point shedders (group B), and 10 OTUs were unique to intermittent/persistent shedder pigs (group C). Operational taxonomic units from *Roseburia*, *Lachnospira*, or *Phascolarctobacterium* were present only in the core microbiome of group A, while the genus *Oscillospira* was present only within the core microbiome of shedding groups B and C.

### Differences in abundance of OTUs between pigs in different categories. (i) Differences according to infection status.

When the fecal microbiota of *Salmonella-*infected and noninfected pigs was compared, we observed differences in abundance of OTUs belonging mainly to the phylum *Firmicutes* but also to *Bacteroidetes* and *Euryarchaeota* (Table 1; Table S4). Most of these OTUs belonged to the class *Clostridia*. An OTU classified within the family *Ruminococcaceae* and another two belonging to the genera *Coprococcus* and *Lachnospira*, both from the family *Lachnospiraceae*, were relatively more abundant (with a >1.5-log fold change) in noninfected pigs.

**TABLE 1:** OTUs found to be differentially abundant in the feces of pigs categorized according to three different variables related to *Salmonella* infection (infection status, serology, and shedding group)^*a*^

Variable	Phylum	Class	Family	Genus	logFC	SD	FDR
Infection status	*Firmicutes*	*Clostridia*	Unclassified	Unclassified	−1.66	0.57	0.010
	*Firmicutes*	*Clostridia*	*Ruminococcaceae*	Unclassified	−1.95	0.79	0.001
	*Firmicutes*	*Clostridia*	*Ruminococcaceae*	Unclassified	2.12	0.88	0.010
	*Firmicutes*	*Clostridia*	*Lachnospiraceae*	*Coprococcus*	1.66	0.79	0.021
	*Firmicutes*	*Clostridia*	*Lachnospiraceae*	*Lachnospira*	1.51	0.56	<0.001
	*Euryarchaeota*	*Methanobacteria*	*Methanobacteriaceae*	*Methanobrevibacter*	1.40	0.63	0.035
	*Bacteroidetes*	*Bacteroidia*	*Paraprevotellaceae*	*Prevotella*	1.34	0.69	<0.001
	*Firmicutes*	*Clostridia*	*Ruminococcaceae*	*Ruminococcus*	1.28	0.58	<0.001
Serology	*Firmicutes*	*Bacilli*	*Lactobacillaceae*	*Lactobacillus*	1.54	0.68	0.040
	*Firmicutes*	*Clostridia*	*Ruminococcaceae*	Unclassified	1.34	0.74	<0.001
	*Bacteroidetes*	*Bacteroidia*	*Prevotellaceae*	*Prevotella*	−1.20	0.54	<0.001
	*Firmicutes*	*Clostridia*	*Ruminococcaceae*	Unclassified	−1.24	0.45	0.031
	*Bacteroidetes*	*Bacteroidia*	*Prevotellaceae*	*Prevotella*	−1.25	0.68	0.039
	*Firmicutes*	*Clostridia*	*Ruminococcaceae*	*Ruminococcus*	−1.27	0.58	<0.001
	*Firmicutes*	*Erysipelotrichia*	*Erysipelotrichaceae*	p-75-a5	−1.30	0.44	0.022
	*Firmicutes*	*Clostridia*	*Ruminococcaceae*	Unclassified	−1.31	0.77	<0.001
	*Firmicutes*	*Clostridia*	*Clostridiaceae*	*Clostridium*	−1.34	0.54	0.019
	*Firmicutes*	*Clostridia*	*Ruminococcaceae*	Unclassified	−1.36	0.45	<0.001
	*Euryarchaeota*	*Methanobacteria*	*Methanobacteriaceae*	*Methanobrevibacter*	−1.36	0.66	<0.001
	*Bacteroidetes*	*Bacteroidia*	*Prevotellaceae*	*Prevotella*	−1.38	0.56	0.009
	*Firmicutes*	*Clostridia*	*Lachnospiraceae*	Unclassified	−1.39	0.57	<0.001
	*Firmicutes*	*Clostridia*	*Lachnospiraceae*	Unclassified	−1.40	0.52	0.022
	*Firmicutes*	*Clostridia*	*Lachnospiraceae*	*Lachnospira*	−1.45	0.64	0.018
	*Bacteroidetes*	*Bacteroidia*	*Prevotellaceae*	*Prevotella*	−1.50	0.50	0.008
	*Firmicutes*	*Clostridia*	*Lachnospiraceae*	Unclassified	−1.51	0.54	0.005
	*Firmicutes*	*Clostridia*	*Veillonellaceae*	*Anaerovibrio*	−1.53	0.68	<0.001
	*Spirochaetes*	*Spirochaetes*	*Spirochaetaceae*	*Treponema*	−1.55	0.79	0.002
	*Firmicutes*	*Clostridia*	*Lachnospiraceae*	*Lachnospira*	−1.60	0.54	0.003
	*Firmicutes*	*Clostridia*	*Ruminococcaceae*	Unclassified	−1.64	0.76	0.031
	*Firmicutes*	*Clostridia*	*Ruminococcaceae*	Unclassified	−2.19	0.68	0.005
Shedding group A and group C	*Firmicutes*	*Bacilli*	*Lactobacillaceae*	*Lactobacillus*	1.68	0.63	<0.001
	*Bacteroidetes*	*Bacteroidia*	*Prevotellaceae*	*Prevotella*	1.66	0.58	<0.001
	*Proteobacteria*	*Gammaproteobacteria*	*Succinivibrionaceae*	*Succinivibrio*	1.64	0.66	<0.001
	*Bacteroidetes*	*Bacteroidia*	*Prevotellaceae*	*Prevotella*	−1.22	0.57	<0.001
	*Firmicutes*	*Erysipelotrichia*	*Erysipelotrichaceae*	p-75-a5	−1.32	0.47	<0.001
	*Firmicutes*	*Clostridia*	*Lachnospiraceae*	Unclassified	−1.36	0.66	0.011
	*Firmicutes*	*Clostridia*	Unclassified	Unclassified	−1.39	0.52	<0.001
	*Firmicutes*	*Clostridia*	*Lachnospiraceae*	Unclassified	−1.40	0.57	<0.001
	*Firmicutes*	*Clostridia*	*Lachnospiraceae*	Unclassified	−1.46	0.61	<0.001
	*Firmicutes*	*Clostridia*	*Ruminococcaceae*	Unclassified	−1.54	0.80	0.003
	*Firmicutes*	*Clostridia*	Unclassified	Unclassified	−1.83	0.58	<0.001
	*Firmicutes*	*Clostridia*	*Ruminococcaceae*	Unclassified	−2.07	1.13	<0.001
	*Bacteroidetes*	*Bacteroidia*	*Prevotellaceae*	*Prevotella*	−2.49	1.22	<0.001

*^a^*Abbreviations: FC, fold change; SD, standard deviation; FDR, false-discovery rate value.

10.1128/mSystems.00021-19.5 TABLE S4 Differentially abundant OTUs associated with *Salmonella* infection assessed according to the three factors included in the study (infection status, serology, and shedding group). Download Table S4, XLSX file, 0.02 MB. Copyright © 2019 Argüello et al. 2019 Argüello et al. This content is distributed under the terms of the Creative Commons Attribution 4.0 International license.

Analysis of infection status-associated abundance differences by sampling time point revealed changes in the genera linked to *Salmonella* infection throughout the study (Table S5). At sampling 1 (weaning period), OTUs in the family *Lachnospiraceae* were associated with noninfected pigs. Similarly, at this time point, the genus *Sutterella* was also more abundant in noninfected pigs, a result which was not observed at subsequent samplings. At the end of the weaning (sampling 2) and growing (sampling 3) periods, OTUs belonging to *Ruminococcus* and *Prevotella* were more abundant in noninfected pigs. Two genera were exclusively more abundant in infected pigs, *Lactobacillus* and *Oscillospira*, both at sampling 2.

10.1128/mSystems.00021-19.6 TABLE S5 Differentially abundant OTUs associated with *Salmonella* infection assessed according to the three factors included in the study (infection status, serology, and shedding group) and sampling time point. Download Table S5, XLSX file, 0.03 MB. Copyright © 2019 Argüello et al. 2019 Argüello et al. This content is distributed under the terms of the Creative Commons Attribution 4.0 International license.

### (ii) Differences according to serological status.

Table 1 lists the OTUs associated with the factor “serological status” (seropositive or seronegative pigs). Most of the OTUs found to be more abundant in seronegative pigs belonged to the phylum *Firmicutes*, in particular to the families *Lachnospiraceae* and *Ruminococcaceae*. *Methanobrevibacter*, *Prevotella, Lachnospira, Anaerovibrio*, and *Ruminococcus* were the genera most frequently linked to the seronegative category, although the latter also had an OTU significantly more abundant in seropositive pigs. In contrast, an OTU from the genus *Lactobacillus* was significantly more abundant (1.5 log) in seropositive than seronegative pigs (Table 1; Table S4).

Analysis of the microbiome by sampling time point revealed shifts in some of the main genera found to be differentially abundant between categories within the variable “serological status.” While *Lachnospira*, *Ruminococcus*, and *Prevotella* OTUs were more abundant in seropositive pigs at the beginning of the study (sampling 1), their abundance shifted at subsequent samplings and we observed significantly higher abundance in seronegative pigs at the end of the weaning (sampling 2) and growing (sampling 3) periods for both genera.

### (iii) Differences associated with *Salmonella* shedding pattern.

**FIG 4:**
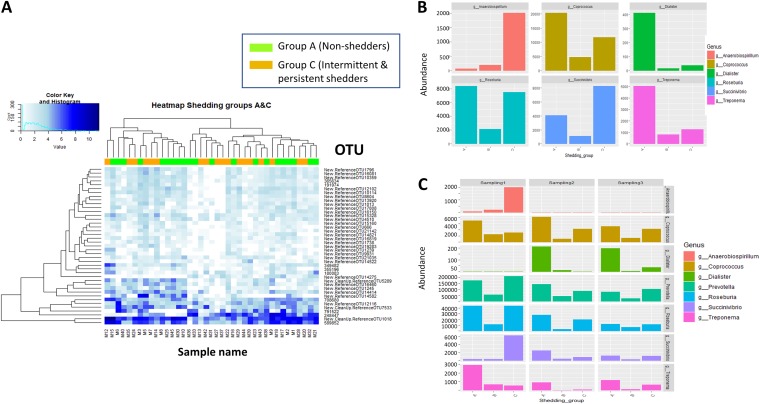
Differences in microbial abundance in weaned pigs categorized according to *Salmonella* shedding. (A) Heat map illustrating the mean counts of differentially abundant OTUs in samples from nonshedder pigs (group A; green) and intermittent/persistent shedder pigs (group C; orange). The white color indicates low-relative-abundance taxa, while dark blue represents those at high relative abundance. The dendrogram was built using hierarchical cluster analysis with Bray-Curtis dissimilarity indices. (B) Abundance of the main genera found to be differentially abundant among shedding groups (A, nonshedders; B, single-point shedders; C, intermittent and persistent shedders). (C) Abundance of the main genera differentially abundant among shedding groups by sampling time point.

Analysis of differences in OTUs among the three shedding groups (group A [nonshedders], group B [single-point shedders], and group C [intermittent and persistent shedders]) revealed four differentially abundant OTUs. However, the limited number of pigs in which these OTUs were present, as well as the low abundances observed, calls into question the relevance of this finding (Table S4). We therefore further analyzed differences using the shedding group variable by limiting the analysis to differences in abundance between groups A and C. No differences in clustering by differentially abundant OTUs were observed between groups A and C (Fig. 4A). In fact, samples from both groups were equally distributed within the two major clades observed in the heat map. However, we did observe particular differences for several taxa. The genera *Lactobacillus* and *Succinivibrio* were more abundant in group C pigs (intermittent/persistent shedders) while OTUs belonging to the families *Lachnospira* and *Ruminococcaceae* were more abundant in group A pigs (nonshedders). Inconclusive results were observed for *Prevotella* OTUs, which were significantly abundant in both groups (Table 1).

Analysis of data by sampling time point (Fig. 4B; Table S5) showed that *Coprococcus* abundance was associated with nonshedder pigs (group A) across all three samplings performed (Fig. 4C). At sampling 1, the genera *Treponema* and *Fibrobacter* were also linked to nonshedders while in subsequent samplings at the end of the weaning and growing stages *Prevotella* and *Dialister* were more abundant in nonshedder pigs. *Lactobacillus* was more abundant at sampling 1 in single-point shedder pigs (group B) as were some OTUs of *Prevotella* at samplings 2 and 3 (Fig. 4C). The order YS2 (*Cyanobacteria*) and the genus *Anaerobiospirillum* were noticeably increased in the intermittent/continuous shedder group (group C). In particular, the genus *Anaerobiospirillum* was increased in abundance in group C at sampling 1 (Fig. 4B).

### Differentially abundant OTUs shared across the *Salmonella* infection-associated variables under study.

Finally, we compared the data sets for the OTUs that were differentially abundant according to the three *Salmonella* infection-associated variables studied. Five OTUs were shared among those differentially abundant according to the variables “infection” and “serological status” (Table 2). It is noteworthy that noninfected and seronegative pigs shared OTUs from the genera *Lachnospira*, *Ruminococcus*, and *Methanobrevibacter*. Similarly, the same OTUs belonging to the families *Lachnospiraceae* and *Ruminococcaceae* and the genus *Prevotella* were present in seronegative and nonshedder (group A) pigs (Table 2). On the other hand, seropositive and intermittent/persistent *Salmonella* shedder (group C) pigs shared a differentially abundant OTU from *Lactobacillus.*

**TABLE 2:** Differentially abundant OTUs shared among variables included in the study: infection, serology, and shedding group

OTU	Taxon	Variable^*a*^		
		Infection	Serology	Shedding group^*b*^
343831	*Clostridiales*	Noninfected	Seronegative	
523140	*Ruminococcus*	Noninfected	Seronegative	
842598	*Methanobrevibacter*	Noninfected	Seronegative	
1029949	*Lachnospira*	Noninfected	Seronegative	
New.Reference OTU2734	*Ruminococcaceae*	Infected	Seropositive	
323200	p-75-a5^*c*^		Seronegative	Group A
339504	*Lachnospiraceae*		Seronegative	Group A
343709	*Ruminococcaceae*		Seronegative	Group A
354905	*Lactobacillus*		Seropositive	Group C
New.Reference OTU14171	*Prevotella*		Seronegative	Group A
New.CleanUp.Reference OTU164624	*Lachnospiraceae*		Seronegative	Group A
New.Reference OTU10282	*Lachnospiraceae*		Seronegative	Group A

a Category in each variable linked to the abundance of the OTU.

b Shedding group category A (nonshedders) and category C (intermittent and persistent shedders).

c Genus belonging to the family *Erysipelotrichaceae*.

## DISCUSSION

The pig gastrointestinal tract is colonized by many different types of microorganisms which contribute to a range of host physiological processes, such as metabolism, integrity of the epithelial barrier, immune homeostasis, and protection against pathogens (18, 20, 26). Competition for niche and nutrients and production of bacteriocins and metabolites are considered the main mechanisms of pathogen exclusion by the resident microbiota (27). Recent studies using 16S rRNA gene sequencing have demonstrated the role of the commensal microbiota in conferring resistance to gut colonization by pathogens (19, 26). In addition, there are already findings from deliberate infection studies in pigs that suggest the influence of microbiota composition on the concentration of *Salmonella* excreted in pig feces (24). Here, for the first time, by parallel monitoring of the *Salmonella* infection and shedding status and microbiome composition of pigs naturally exposed to *Salmonella* under field conditions, we aimed to identify microbial communities associated with infection susceptibility. Disease susceptibility was defined by the combination of data, shedding of the pathogen in feces, and development of specific anti-*Salmonella* antibodies, together with three potential shedding scenarios. It was anticipated that the combination of this information and microbiome data would provide insight into the differences in infection outcome observed among individuals, thereby informing new control strategies for *Salmonella* in pigs.

### Microbiome diversity may prevent early infection of pigs postweaning.

The outcome of *Salmonella* infection in pigs is usually evaluated through direct methods which include microbiological detection of the bacterium and indirect methods which look for markers such as antibodies within the host (14). The two methods complement each other and were used in the present study to categorize pigs according to their infection outcome. Interestingly, microbiome diversity and ordination were associated with differences in the serological status of the animals. Two weeks after weaning, diversity of the fecal microbiota was higher in seronegative pigs, i.e., those without antibodies (anti-LPS IgG) to the pathogen. Two factors increase disease susceptibility postweaning: (i) the transition from milk to a solid diet, which shifts the microbial composition of the gut toward what is often considered a transitory dysbiosis (28); and (ii) the loss of maternal protective immunoglobulins provided by sows’ milk (29). Therefore, taking our results and the first factor above into consideration, early establishment of a diverse and healthy microbiota may hamper the colonization success of pathogens such as *Salmonella.* The suggestion that gut health correlates with microbiome richness is in line with previous studies (30, 31). This is further supported by our study, which also showed an association between serological status and microbial ordination. From our results, we can infer that a more complex early-life microbiome may provide a more challenging environment for pathogens such as *Salmonella*, limiting their infective capacity and thereby preventing intestinal invasion and activation of the humoral immune response (32).

### Members of the class *Clostridia* may prevent colonization of the gastrointestinal tract by *Salmonella*.

Members of the class *Clostridia*, including the genera *Roseburia* and *Blautia* from the family *Lachnospiraceae* and the genera *Ruminococcus* and *Anaerovibrio*, were more abundant in *Salmonella*-negative pigs. Furthermore, *Roseburia* and *Blautia* (*Lachnospiraceae*) together with the genus *Phascolarctobacterium* were part of the core microbiome of noninfected pigs. Previous studies link these taxa to a healthy gut configuration in mammals (25, 33–35). In addition, metagenomic studies have found a negative correlation between anaerobe counts and epithelial damage in the ileal mucosa (25) and a higher abundance of *Ruminococcaceae* prior to challenge in pigs shedding low *Salmonella* concentrations (24). Another common feature of these genera is that they are preferentially or strict anaerobes and producers of short-chain fatty acids (SCFA) (33, 35–37). These two factors, together with limited oxygen and high concentrations of SCFA, such as butyrate, prevent the expansion of facultatively anaerobic Enterobacteriaceae such as *Salmonella* (38). The dominance of anaerobes in *Salmonella*-negative pigs is supported by the higher counts of the genus *Methanobrevibacter* in seronegative pigs. Methanogens such as *Methanobrevibacter* obtain energy from hydrogen molecules produced by strict anaerobes (39), and their abundance is linked to the presence of these anaerobes (20). In contrast, *Salmonella*-infected pigs had higher counts of *Lactobacillus and Oscillospira* OTUs. *Lactobacillus* and *Oscillospira* are both characteristic of the gut microbiome of nursing pigs (40, 41). We propose that their presence in high counts in feces 18 days pw (∼6 weeks of age) could be indicative of immaturity of the microbiota and a lack of commensal organisms that restrict *Salmonella* colonization. Another potential explanation could be the presence of more favorable conditions for *Lactobacillus* growth in infected pigs, a result already reported by Drumo and colleagues (42). However, the mechanisms by which *Salmonella* infection might boost the growth of these taxa remain unknown.

### Changes in the competitive microbiota throughout different life stages of the pig.

Under intensive production conditions, the microbiota of the pig gastrointestinal tract evolves, shifting from *Bacteroidetes* to *Firmicutes* as the animal grows (40, 43). Putative components of the microbiota which participate in competitive exclusion of pathogens may also vary from one life stage to another. In our study, we also observed a strong effect of sampling time point on microbiome composition. Thus, sampling time point was first used as a cofactor in the statistical analyses, and then longitudinal differences in microbiota abundance were analyzed over time. Early after weaning (18 days pw; ∼6 weeks of age), we observed a higher abundance of the genus *Sutterella* in noninfected pigs. *Sutterella* is a member of the *Proteobacteria* which predominantly inhabits the small intestine, at least in humans (44). Although its role in intestinal health is unclear, it is considered a commensal, is capable of adhering to the epithelium, and has a mild proinflammatory capacity. Members of this genus could therefore potentially occupy the niche of pathogenic *Proteobacteria* or positively stimulate the immune response, although more research is needed to explore this. In samplings at the end of the weaning (∼8 weeks of age) and growing (∼11 weeks of age) periods, OTUs of *Ruminococcus* and *Prevotella* were enriched in the noninfected pigs. Both genera are cellulolytic microbiota that increase in abundance during maturation of the gut microbiota in pigs (40, 45). These two genera were also part of the healthy gut configuration in patients who had recovered from Vibrio cholerae infection (46, 47), adding further support to the theory proposed in the present study that anaerobic cellulolytic SCFA-producing bacteria limit the success of *Salmonella* in colonizing the pig gastrointestinal tract.

### Persistence of *Salmonella* shedding may be influenced by the presence of synergistic bacteria.

The final goal of the present study aimed to investigate the role of the gut microbiome in determining the distinct shedding patterns observed in the animals under study. After an acute phase of infection, characterized by continuous shedding of high concentrations of *Salmonella* (48), nontyphoidal *Salmonella* infection in pigs progresses to a chronic phase, with no evident clinical signs and intermittent shedding of the pathogen in feces. This is a result of the combination of lower concentrations of *Salmonella* in feces and the limitation of microbiological methods to detect this low burden of *Salmonella* (49). By monitoring naturally infected pigs throughout the weaning and growing periods, we observed differences in *Salmonella* shedding patterns from single-point shedder pigs to a pig which was positive at all five samplings performed. Although clustering analysis by abundance of OTUs did not split samples according to the shedding group, there were differences in particular taxa. In addition to higher abundance of *Lactobacillus*, both in single-point and in intermittent/continuous shedder groups, we observed another two taxa that were overrepresented in these groups: the genus *Anaerobiospirillum* in single-point shedder pigs and the order YS2 in intermittent/continuous shedder pigs. *Anaerobiospirillum* can be considered a pathobiont, an indigenous microbe that is able to promote disease under certain circumstances (18, 50), and its presence has been linked to diarrhea in humans (51, 52). However, little is known about YS2, an order included in the phylum *Cyanobacteria*, and the reason why it is more abundant in the gastrointestinal tract of pigs shedding *Salmonella* is unclear.

### Conclusion.

Field studies allow the investigation of diseases under natural conditions, revealing interesting information which may not come to light in challenge studies. The present field study is the first in which *Salmonella* colonization resistance was studied in pigs. Although the study includes a limited number of animals, the information that they provide is of potentially great value. Our results suggest that early establishment of a diverse core microbiome enriched in anaerobes capable of producing SCFA metabolites and subsequent enrichment of cellulolytic bacteria may impede *Salmonella* gut colonization and invasion and limit fecal shedding. On the other hand, a lack of maturation of the microbiome, with a predominance of microorganisms normally associated with suckling, may increase susceptibility to infection and persistence of pathogen shedding in the feces. Overall, these results suggest that certain taxa within the porcine intestinal microbial community could potentially be targeted in the future to manipulate the intestinal microbiome so as to increase resistance to infection with *Salmonella* in pigs.

## MATERIALS AND METHODS

### Study design.

The study was conducted under license from the Department of Health and Children (number B100/2982) and received ethical approval from the University College Dublin Animal Research Ethics Committee (AREC 13-37).

**FIG 5:**
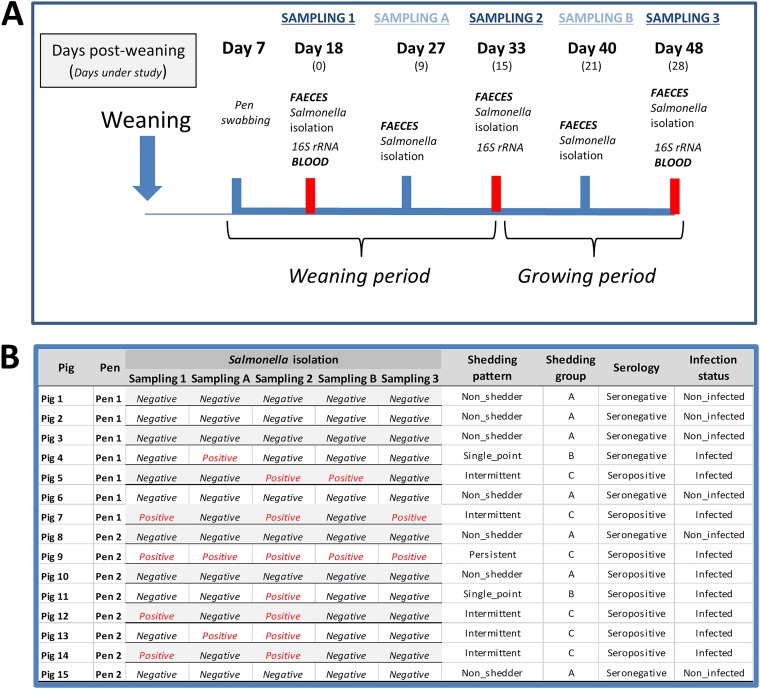
Study design. (A) Samples collected from weaned pigs naturally infected with *Salmonella* or noninfected during a field study conducted on a commercial pig farm. (B) *Salmonella* status of pigs included in the study. *Salmonella* isolation results from fecal samples (ISO 6579/2007) and serology results from anti-*Salmonella* IgG detection (ELISA) in serum of the monitored pigs. Categorization into groups by “shedding pattern”: (i) nonshedders (*Salmonella* negative at all samplings), (ii) single-point shedders (*Salmonella* positive once), (iii) intermittent shedders (*Salmonella* positive between two and four times), and (iv) persistent shedders (*Salmonella* positive at all samplings). These shedding patterns were grouped into three “shedding groups”: group A (nonshedders), group B (single-point shedders), and group C (intermittent and persistent shedders). Serology cutoff was set at 40% of ELISA optical density. Finally, pigs were categorized by “infection status,” with noninfected pigs referring to those that were nonshedders as well as seronegative and infected referring to the other combinations.

The study was performed on an Irish 750-sow commercial farrow-to-finish farm with endemic *Salmonella* (*S. Typhimurium*) infection. One batch of 15 pigs was monitored from approximately 2 weeks after weaning (18 days pw or∼45 days of age) to the end of the growing phase (48 days pw or ∼75 days of age) (Fig. 5). Seven days after pigs were transferred to the weaning facility, eight pens were swabbed using sponges, as previously described (53), and tested for the presence of *Salmonella,* as outlined below. Pigs from two *Salmonella-*positive pens (7 pigs from one pen and 8 from the other) were ear tagged for identification purposes and fecally sampled by digital rectal stimulation 18 days pw (sampling 1) and on four further occasions during the weaning and growing periods (Fig. 5). All fecal samples were tested for the presence of *Salmonella* as outlined below, while for microbiota analysis a subsample was flash-frozen in dry ice at sampling 1, sampling 2, and sampling 3 and stored at −80°C until analysis. Blood samples were collected at the beginning (sampling 1) and end (sampling 3) of the study by jugular venipuncture using whole-blood plastic Vacutainers (BD Vacutainer; Becton Dickinson, Oxford, United Kingdom) for the detection of *Salmonella* antibodies, as outlined below. Throughout the course of the study, none of the study animals received antibiotics or displayed clinical signs compatible with *Salmonella* infection or any other disease of pigs. Animals received two different diets during the weaning and growing stages, respectively.

### *Salmonella* detection in pen swabs and feces.

All pen swabs and fecal samples were tested for the presence of *Salmonella* according to Annex D of the ISO 6579 method, as previously described (53). Based on the results from the fecal samples, pigs were classified into four “shedding patterns” (Fig. 5): (i) nonshedders (7 pigs), defined as animals which were *Salmonella* negative at all samplings; (ii) single-point shedders (2 pigs), pigs which shed *Salmonella* at only one of the sampling time points; (iii) intermittent shedders (5 pigs), defined as pigs which shed *Salmonella* at between two and four of the sampling time points; and (iv) persistent shedders (1 pig), which were pigs that shed *Salmonella* at all samplings. These shedding patterns were grouped into three “shedding groups,” group A (nonshedders), group B (single-point shedders), and group C (intermittent and persistent shedders), for statistical analysis of microbiome differences.

### *Salmonella* serological analysis and definition of infected/noninfected pigs.

Serum was obtained from the blood samples and analyzed in duplicate using an in-house indirect enzyme-linked immunosorbent assay (ELISA) as previously described (53). Briefly, the method detects immunoglobulin G against the O side chain of the lipopolysaccharide of *Salmonella* (54). Optical density percentages (OD%) were determined by relating each serum absorbance value at 650 nm to that of the positive control. According to the ELISA results, pigs were defined as seropositive (8 pigs) when the ELISA OD value was ≥10% and seronegative (7 pigs) when the OD value was <10% (Fig. 5).

By combining the *Salmonella* shedding and serological data, pigs were defined by a new variable referred to as “infection status”: “noninfected” pigs (6 pigs) were defined as those which were bacteriologically and serologically negative on all occasions, while “infected” (9 pigs) comprised all other pigs which were *Salmonella* positive in the feces, seropositive, or both.

### 16S rRNA amplicon sequencing of fecal microbiota.

Total DNA was extracted from all fecal samples (∼200 mg) using the QIAamp DNA stool minikit (Qiagen, Crawley, United Kingdom) according to the manufacturer's instructions apart from adding a bead-beating step after sample addition to the InhibitEX buffer and increasing the lysis temperature to 95°C to increase the DNA yield (23). All samples were prepared for MiSeq compositional sequencing using the specifications outlined by Illumina (Illumina Inc., Cambridge, United Kingdom). The V3-V4 region of the 16S rRNA gene was amplified, and Illumina index primers was attached in two separate PCRs (55). All PCR conditions and cleanup procedures using AMPure XP (Labplan, Kildare, Ireland) were as outlined by Illumina. Quantified samples were then sequenced using an Illumina MiSeq system at the Teagasc Sequencing Centre (Fermoy, Ireland).

### Bioinformatic processing and analysis.

Raw sequence reads generated by MiSeq were processed using version 1.9.1 of the Quantitative Insights Into Microbial Ecology (QIIME) pipeline (56) by using the subsampled open-reference OTU calling approach (57). Demultiplexing and trimming of MiSeq reads were performed using the default QIIME parameters (58). After trimming, the reads were merged into a single FASTA file and clustered into OTUs against the Greengenes database (59) (release 2013-08; gg_13_8_otus) by using the parallel *uclust_ref* method. Reads that failed this step were clustered into *de novo* OTUs using the *uclust* method (60). The filtering of chimeric OTUs was performed using ChimeraSlayer (61) against the Greengenes reference alignment. After removing singleton and doubleton OTUs, only those OTUs representing >0.005% of the total filtered were retained as suggested by Bokulich et al. (58). For analysis at the genus level, OTUs were collapsed into genus taxonomic level using the tax_glom function in Phyloseq (62).

### Statistical analysis.

Statistical analyses were performed in R v3.4.2. Microbiota and study variables (shedding pattern, shedding group, serology, infection status, pen, and sampling time point) were included in the estimation of alpha-diversity richness (Shannon, Simpson, and Chao1 indices) by the Nmle, Vegan, and Phyloseq R packages (62, 63). For richness values, assumption of normality was checked using the Shapiro-Wilk test, and potential differences in richness of factors included in the study were estimated by repeated-measures analysis of variance (ANOVA), using either sampling time point or pig as a cofactor and a Tukey multiple-comparison test. Dissimilarities in beta-diversity between pairs of samples were estimated with the Bray-Curtis dissimilarity index (64) and weighted UniFrac index (65) and analyzed with nonlinear multidimensional scaling (NMDS) in Vegan. The Vegan *envfit* function, which fits environmental vectors or factors onto an ordination, was used to evaluate if the factors sampling day and infection status were associated with the NMDS ordinations; the significance of the fitted factors was estimated using 999 permutations. Core microbiome was established as those OTUs present in ≥50% of the samples and presenting a mean relative abundance of >1% within the corresponding groups, using the core function in the R package Microbiome (66). Differences in taxon abundance were analyzed after OTU count normalization by calculating the scaling factors equal to the sum of counts in the metagenomeSeq R package (67). The zero-inflated, log-normal distribution (*fitFeatureModel* function) and the zero-inflated Gaussian distribution mixture-model (*fitZig* function) were used to estimate differences in variables under study, using sampling time point and pig factors as covariates and with a false-discovery rate (FDR) threshold of 0.05.

### Accession number(s).

The full data sets have been submitted under BioProject accession no. PRJNA521510.
