# Supplementary figures and images for: Influence of the Intestinal Microbiota on Colonization Resistance to Salmonella and the Shedding Pattern of Naturally Exposed Pigs
Source: mSystems. 2019 Apr 23;4(2):e00021-19. doi: 10.1128/mSystems.00021-19 (PMC6478965; doi:10.1128/mSystems.00021-19)

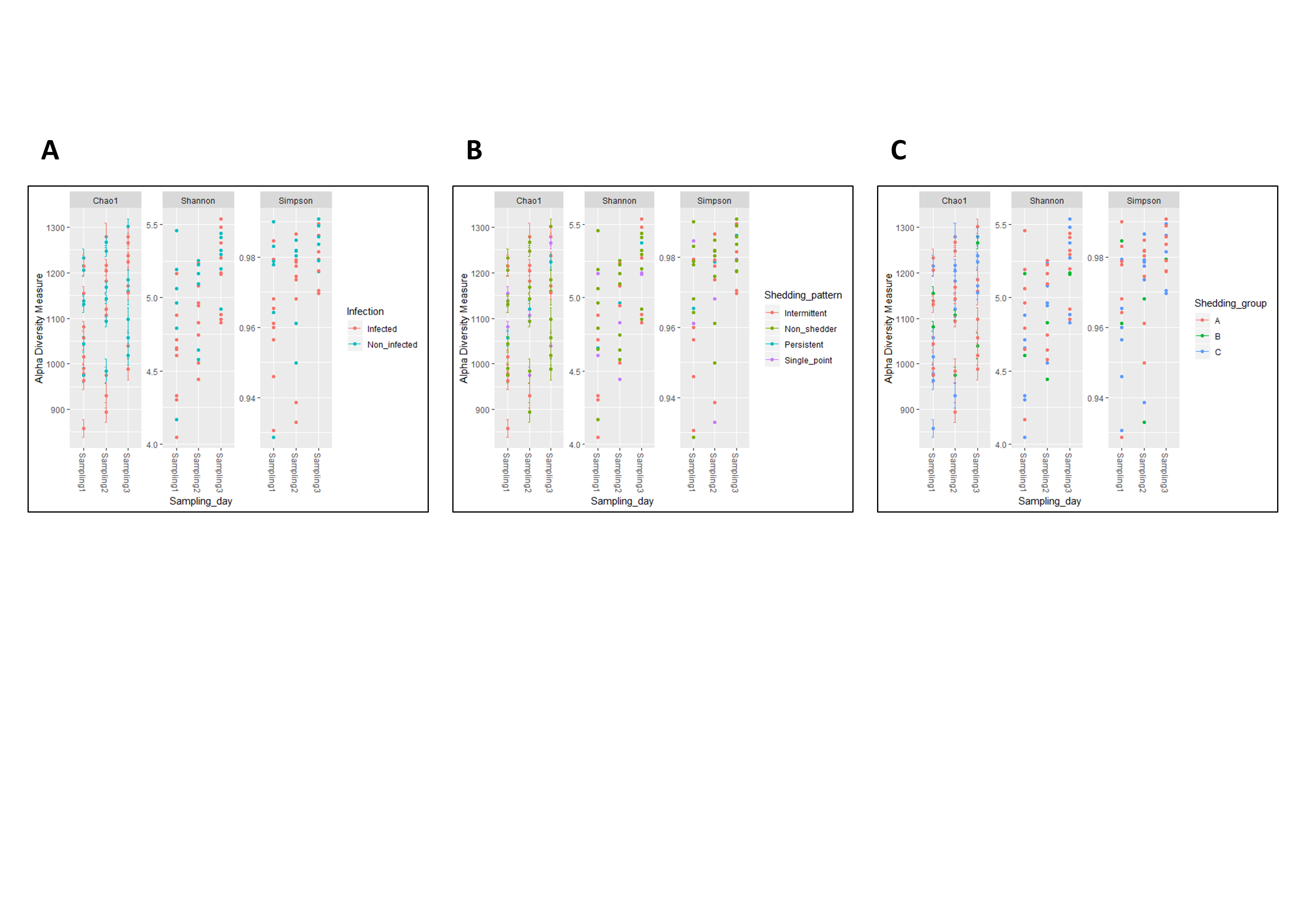

Supplement: FIG S1 [file mSystems.00021-19-sf001.tif]
